# High But Not Low Probability of Gain Elicits a Positive Feeling Leading to the Framing Effect

**DOI:** 10.3389/fpsyg.2017.00081

**Published:** 2017-02-09

**Authors:** Corentin J. Gosling, Sylvain Moutier

**Affiliations:** Laboratory of Psychopathology and Health Processes (EA 4057), Department of Psychology, Paris Descartes University, Sorbonne Paris City UniversityParis, France

**Keywords:** framing effect, emotion, decision-making, loss aversion, risk-seeking, gain anticipation

## Abstract

Human risky decision-making is known to be highly susceptible to profit-motivated responses elicited by the way in which options are framed. In fact, studies investigating the framing effect have shown that the choice between sure and risky options depends on how these options are presented. Interestingly, the probability of gain of the risky option has been highlighted as one of the main factors causing variations in susceptibility to the framing effect. However, while it has been shown that high probabilities of gain of the risky option systematically lead to framing bias, questions remain about the influence of low probabilities of gain. Therefore, the first aim of this paper was to clarify the respective roles of high and low probabilities of gain in the framing effect. Due to the difference between studies using a within- or between-subjects design, we conducted a first study investigating the respective roles of these designs. For both designs, we showed that trials with a high probability of gain led to the framing effect whereas those with a low probability did not. Second, as emotions are known to play a key role in the framing effect, we sought to determine whether they are responsible for such a debiasing effect of the low probability of gain. Our second study thus investigated the relationship between emotion and the framing effect depending on high and low probabilities. Our results revealed that positive emotion was related to risk-seeking in the loss frame, but only for trials with a high probability of gain. Taken together, these results support the interpretation that low probabilities of gain suppress the framing effect because they prevent the positive emotion of gain anticipation.

## Introduction

Choosing between several options is a major challenge in our everyday lives. As we operate with limited resources, our decisions systematically require an evaluation of energy costs and potential rewards (Rangel et al., 2008). This evaluation enables us to select options that minimize costs and maximize benefits, i.e., leading to the best outcomes in terms of biological fitness. Therefore, the ability to predict future outcomes of decisions, in order to confirm or cancel the action according to environmental modifications, is central in decision-making (Mirabella, 2014).

Although human decision-making has been typically described as perfectly rational, over the last three decades, numerous studies have indicated that individuals often deviate from predictions based on rational choice theory (Reyna and Ellis, 1994; Kahneman, 2003; Evans, 2008; Cassotti and Moutier, 2010). Many authors have postulated that decisional biases arise from a competition between two distinct types of reasoning, i.e., between an intuitive-heuristic form of reasoning – Type 1 – and an executive-analytic form of mental operations – Type 2 (Sloman, 1996; De Neys, 2006; Evans, 2011; Kahneman, 2012). Type 1 processing operates quickly, is effortless, independent of working memory and cognitive ability. Everyday mental activities, such as knowing that 2+2 = 4 or thinking of London when the capital of Great Britain is mentioned, are some examples of the automatic activities that are attributed to Type 1 thinking. On the other hand, Type 2 processing is relatively slow, effortful, heavily dependent on working memory and related to individual differences in cognitive ability (Evans, 2011). For example, Type 2 thinking is credited with the continuous monitoring of our own behavior – the control that keeps us polite when we are angry or alerts when we are driving at night. However, continuous vigilance is certainly impractical. Because Type 1 is fast, errors of intuitive thought are often difficult to prevent and cannot be always filtered by Type 2.

Tversky and Kahneman (1981) were among the first to highlight that decisions adults make could break logico-mathematical rules. They showed that a choice between two options could vary depending on how these options are presented, despite their strict mathematical equality. They named this cognitive bias the framing effect. According to Kahneman and Frederick (2007), the framing effect occurs because an emotional heuristic belonging to Type 1 thinking leads to a shift in preferences according to the formulation of options (intuitive-heuristic behavior), thereby violating the invariance principle (analytic behavior). This emotional heuristic rises from a strong attractiveness of the sure gains, as well as a high aversion of the sure losses. Recent neuroimaging and behavioral studies have provided evidence in support of this assumption (De Martino et al., 2006; Cheung and Mikels, 2011; Cassotti et al., 2012).

In their initial paradigm used to assess framing susceptibility (the “Asian-disease problem”; Tversky and Kahneman, 1981), participants were presented with a choice between a sure and a risky option with identical expected values. Interestingly, when the choice was framed in terms of gain, participants tended to choose the sure option (“200 people will be saved”) more frequently than the risky option (“1/3 probability that 600 people will be saved, and 2/3 probability that no one will be saved”). In contrast, when the choice was framed in terms of loss, they tended to choose the risky option (“2/3 probability that 600 people will die, and a 1/3 probability that no one will die”) more frequently than the sure option (“400 people will die”). This type of framing effect, named “the risky choice framing effect” (Levin et al., 1998), is the gold standard, and thus we exploited it.

Following the initial work of Tversky and Kahneman (1981), several studies focused on factors that moderate susceptibility to the framing effect. First, probability of gain of the risky option was underlined as one of the main factors causing variations in this susceptibility (Hogarth and Einhorn, 1990; Tversky and Kahneman, 1992). In fact, while high probabilities of gain lead to the framing effect, low probabilities of gain could suppress susceptibility to it (Kühberger et al., 1999). Second, as previously mentioned, many studies have emphasized the role of emotions in the framing effect (De Martino et al., 2006; Cheung and Mikels, 2011; Cassotti et al., 2012). Until now, no research has considered these two factors. Thus, as emotions play a key role in the emergence of the framing effect, do they underpin its suppression when low probabilities of gain are at play?

Shortly after the initial description of the framing effect by Tversky and Kahneman (1981), the Venture Theory (Hogarth and Einhorn, 1990) showed that a higher probability of gain was associated with increased risk-aversion in the gain frame and with increased risk-seeking in the loss frame. In other words, participants were more susceptible to the framing effect when probability of gain was high rather than low. Moreover, Tversky and Kahneman (1992) proposed an alternative to the Venture Theory called the Cumulative Prospect Theory. The Cumulative Prospect Theory predicted that for high probabilities of gain, individuals would show the classic framing effect. In contrast, this theory predicted that for low probabilities of gain, individuals would show a reverse framing, i.e., would be more risk-seeking in the gain frame than in the loss frame. For high probabilities of gain, these two theories thus predicted identical decision-making. However, for low probabilities of gain, while the Cumulative Prospect Theory predicted reverse framing, the Venture Theory predicted a reduced framing effect. To decide between these two theories, a meta-analysis on Asian-disease-type experiments was carried out (Kühberger et al., 1999). It revealed that participants demonstrated a stronger framing effect for high probabilities of gain than for low ones. However, for low probabilities of gain, participants did not demonstrate reverse framing. These results therefore reinforced the Venture Theory but contradicted the Cumulative Prospect Theory.

In addition, Kühberger et al. (1999) conducted an experimental study focusing on the influence of probability of gain on the framing effect. This study demonstrated a framing effect for trials with a high probability of gain, but not with a low probability of gain. It is important to note that the Asian-disease-type experiments selected in Kühberger’s et al. (1999) meta-analysis classically used a between-subjects design whereas in their experimental study, these authors used a within-subjects design. As it has been shown that a within-subjects design could decrease framing susceptibility compared to a between-subjects design (Levin et al., 1987), a likely explanation of the difference between the meta-analysis and the experimental study of Kühberger et al. (1999) lies in the type of design used.

Besides, in order to investigate the origins of the framing effect, De Martino et al. (2006) designed a new framing task and examined the relationships between framing susceptibility and brain regions activation. In each trial of this task, participants first received a certain amount of money to play in that trial. Then, they were told that they would not be able to keep this entire initial amount and had to choose between a sure and a risky option of equally expected value within 4 s. The sure option was framed in terms of either gain or loss. Their results showed that decisions congruent with the framing effect were associated with greater bilateral amygdala activation, a brain area known to be involved in emotional processes (Adolphs et al., 1994). This study was one of the first to highlight the key role of emotions in the framing effect. Based on these data, Kahneman and Frederick (2007) suggested that the framing effect could arise from two emotional mechanisms. They hypothesized that, compared to an equivalent risky option, a sure gain is emotionally attractive whereas a sure loss is emotionally aversive.

To our knowledge, only one study has focused on the link between self-reported emotion and the framing effect (Cheung and Mikels, 2011). Using the same task as De Martino et al. (2006), Cheung and Mikels (2011) measured the valence of participants’ emotion about their choices. Results showed that the risky choice is associated with positive emotion for trials in the loss frame but not in the gain frame. This could suggest that in the loss frame, risk-seeking participants felt positive emotions because the risky choice allowed them to anticipate a gain, while the sure choice provided a sure loss. This interpretation is supported by the fact that participants’ risk propensity decreases in the loss frame when facing a positive emotional context before each trial (Cassotti et al., 2012). In fact, the induction of this first positive emotional context probably decreases the effect of the second positive emotion, i.e., gain anticipation, leading to decrease their risk propensity. Therefore, the probability of gain of the risky option may have an influence on the positive emotional mechanism of gain anticipation and thus on the likelihood of engaging in risky choices in the loss frame. Thus, low probabilities of gain should prevent participants from anticipating a gain, leading to a suppression of the framing effect.

The aim of this paper was to clarify the influence of the probability of gain on the framing effect and, for the first time, to provide an explanation for such an influence. Our first study examined this influence by comparing between- and within-subjects designs. Then, our second study aimed to examine the role of the probability of gain in the relationship between emotions and risk-seeking in the framing effect.

## Study 1

In the first study, we aimed to determine whether the influence of the probability of gain on the framing effect was due to the experimental design. In agreement with most studies on the framing effect (Kühberger, 1998), we expected that, overall, participants would show a framing effect. Furthermore, we predicted that in the between-subjects design, participants would be more susceptible to the framing effect for the high compared to the low probability condition, but we expected that participants would still be affected by framing effect in the low probability condition (Hogarth and Einhorn, 1990). In the within-subjects design, we predicted that only trials with a high probability of gain would lead to a framing effect (Kühberger et al., 1999).

### Method

#### Participants

Ninety-six undergraduate students (48 women, mean age = 21.10, *SD* = 2.66) from the University of Paris VIII Saint-Denis were randomly assigned to four experimental groups. Our studies were approved by the University ethical review board and all participants gave their written consent prior to the investigation, in accordance with the Declaration of Helsinki.

#### Material and Procedure

Participants carried out a computerized financial decision-making task created by Cassotti et al. (2012), which was similar to that of De Martino et al. (2006). The task was presented on a 15-in. laptop with E-Prime^®^ experimental software (Psychology Software Tools, Inc., Sharpsburg, PA, USA) at a viewing distance of approximatively 60 cm. This task included four trials according to a 2 (Frame: Gain vs. Loss) × 2 (Probability of gain: Low 30% vs. High 70%)^1^ within-subjects design. Across groups, participants completed the same four trials, i.e., two trials in the gain frame, one with a low and one with a high probability of gain, and two trials in the loss frame, one with a low and one with a high probability of gain. However, these trials were presented in a different order to the different groups (see **Table 1**). By manipulating the presentation order, our objective was that each of the four trials was presented as the first trial for one group. Therefore, comparing the first trial carried out by participants of the four groups allowed us to investigate the influence of the probability of gain on the framing effect in a between-subjects design. Across groups, participants always started with two trials in the same frame and then carried on with two trials in the other frame. Probability of gain alternated between each trial.

**Table 1:** Presentation order of the four trials according to the group.

	Presentation order of the four trials (Frame – Probability of gain)			
	1	2	3	4
Group 1	Gain – Low	Gain – High	Loss – Low	Loss – High
Group 2	Gain – High	Gain – Low	Loss – High	Loss – Low
Group 3	Loss – Low	Loss – High	Gain – Low	Gain – High
Group 4	Loss – High	Loss – Low	Gain – High	Gain – Low

**FIGURE 1:**
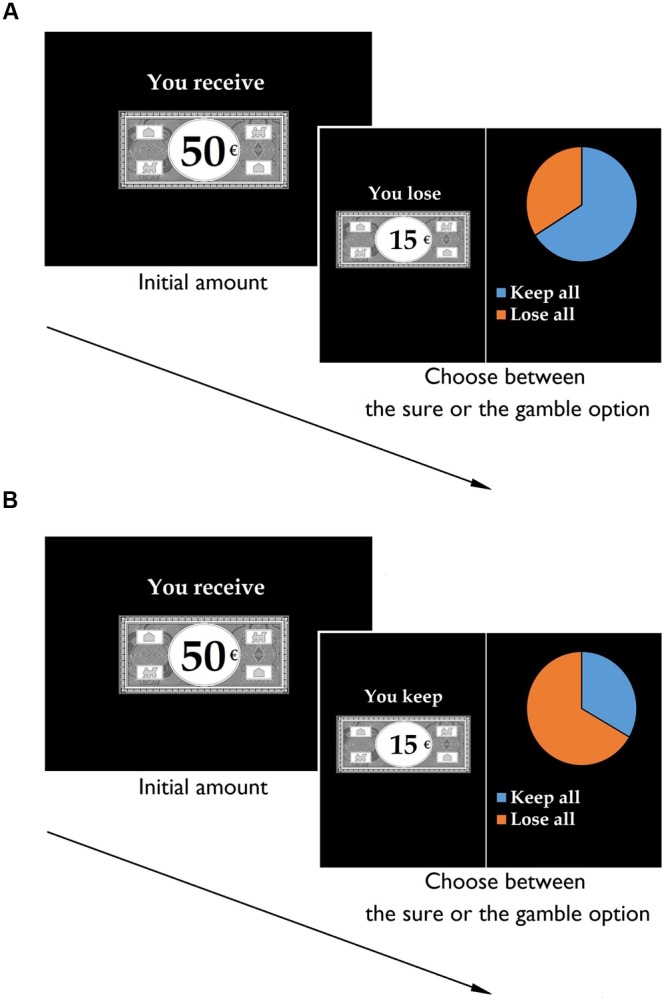
**The financial decision-making task.** Participants were told that they would first receive an initial amount. Second, they were instructed that they would not be able to retain the entire initial amount and would have to chose between a sure and a risky option. Example of a loss trial with a high probability of gain **(A)** and a gain trial with a low probability of gain **(B)**.

For each trial, participants received an initial amount of 50€, presented for 2500 ms. They were then given a choice between two options (see **Figure 1**). A sure option, represented by a bank note, allowed them to keep part of the initial amount while a risky option, represented by a pie chart, allowed them to gamble the initial amount. By selecting the risky option, they had a probability of keeping or losing the entire initial amount. The expected values across options and frames were systematically identical. The formulation of the sure option varied according to the frame. In the gain frame, participants were told that they would keep part of the initial 50€ (e.g., “you keep 15€”), whereas in the loss frame, they were told that they would lose part of the initial 50€ (e.g., “you lose 35€”). In contrast, the risky option was identical in the gain and loss frames. Regardless of which option the participants chose, no feedback was supplied.

Before starting this task, instructions were given through a practice trial in which the amount and the frame of the sure option was not specified and the wheel of fortune depicting the probability of gain was empty (see supplementary material). This instruction design was used to ensure that the frame, the sure amount or the probability of gain of the practice trial would not influence participants.

Choice of the risky option was coded 1 and choice of the sure option was coded 0. For the purpose of clarity, results are presented as percentages of risky choices. Data were processed using SPSS v20 (SPSS Inc., Armonk, NY, USA). We carried out an analysis of variance (ANOVA; Lunney, 1970; D’Agostino, 1971), followed by comparisons of means (Tukey’s Honestly Significant Difference). Effect sizes were measured using partial eta-squared.

### Results

Results of this first study are presented in two steps: first, according to the between-subjects design (i.e., within the first trial of each group) and second, according to the within-subjects design (i.e., across all trials carried out by the participants).

First, to study the effect of the probability of gain on the framing effect while avoiding any comparison effect between trials, we conducted a two-way ANOVA on the first trial with Frame (Gain vs. Loss) and Probability of gain (Low vs. High) as between-subjects factors. A significant main effect of Frame revealed that participants were overall susceptible to the framing effect [*F*(1,92) = 5.60; *p* < 0.05; η_p_^2^ = 0.06], in that they made more risky choices in the loss frame (*M* ±*SD*; *M*_Loss_ = 60% ± 49) than in the gain frame (*M*_Gain_ = 38% ± 49). A significant interaction was found between Frame and Probability of gain [*F*(1,92) = 7.82; *p* < 0.01; η_p_^2^ = 0.08], indicating that participants demonstrated a framing effect in the high probability condition (*p* < 0.01; *M*_Loss_ = 79% ± 41; *M*_Gain_ = 29% ± 46; η_p_^2^ = 0.25) but not in the low probability condition (*p* > 0.95; *M*_Loss_ = 42% ± 50; *M*_Gain_ = 46% ± 51).

**FIGURE 2:**
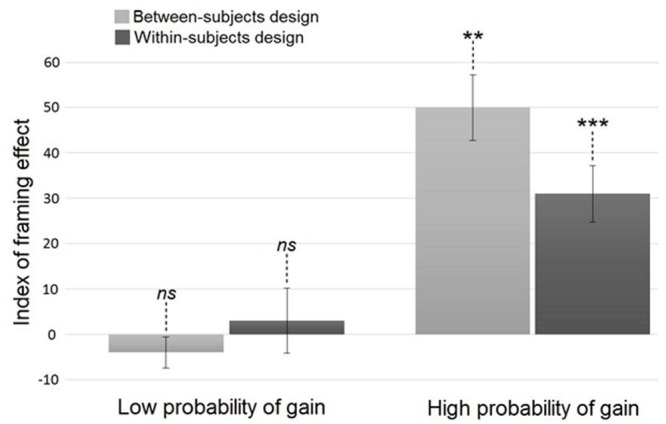
**Index of the framing effect according to the probability of gain and the within/between-subjects design.** ^∗∗^*p* < 0.01; ^∗∗∗^*p* < 0.001; ns, not significant. Error bars: ± Standard Error of the Mean.

Second, to study the influence of the probability of gain on the framing effect while allowing an effect of the comparison between trials, we conducted a three-way ANOVA on all trials with Order as between-subjects and Frame and Probability of gain as within-subjects factors. Neither the main effect of Order [*F*(3,92) = 0.23; *p* > 0.85], nor the Order × Frame [*F*(3,92) = 1.10; *p* > 0.35], nor the Order × Frame × Probability of gain interactions [*F*(3,92) = 1.12; *p* > 0.30] were significant. Besides, we found a significant main effect of Frame [*F*(1,95) = 15.93; *p* < 0.001; η_p_^2^ = 0.14] in that participants made more risky choices in the loss frame (*M*_Loss_ = 60% ± 32) than in the gain frame (*M*_Gain_ = 43% ± 31). As in the previous analysis, the Frame × Probability of gain interaction was significant [*F*(1,95) = 10.70; *p* < 0.01; η_p_^2^ = 0.10], indicating that participants demonstrated a framing effect in the high probability (*p* < 0.001; *M*_Loss_ = 74% ± 44; *M*_Gain_ = 43% ± 50; η_p_^2^ = 0.22) but not in the low probability condition (*p* > 0.90; *M*_Loss_ = 47% ± 50; *M*_Gain_ = 44% ± 50; see **Figure 2**). The index of framing effect presented in **Figure 2** is the percentage difference between the frequency with which subjects chose the risky option in the loss and gain frames (Index value = M_Loss_ - M_Gain_). A positive index value indicates an increase in gambling in the loss compared to the gain frame, i.e., a framing effect. Therefore, the higher the value of this index, the stronger the framing effect.

## Study 2

Our first study confirmed that the framing effect, known as one of the most striking cognitive biases in financial decision-making, could quite easily be canceled through manipulation of the probability of gain. In this second study, in order to determine whether emotions are responsible for such variations of the framing effect according to the probability of gain, we investigated the relationship between participants’ emotions and their choices. Literature on this topic reports three major types of emotions that could influence decision-making (Loewenstein and Lerner, 2003). First, decision makers could be influenced by emotions that they anticipate feeling when the outcome occurs, what the authors call expected emotions. These are directly linked to the expected consequences of decisions. The second type of emotions influencing decisions are incidental emotions, and would be aroused by background factors unrelated to the decision. Finally, at the time of decision, decision makers would feel immediate emotions, which mainly result from the combined effect of expected and incidental emotions. Therefore, in order to study the impact of the emotional context on the framing bias, we chose to focus on the relationship between immediate emotions and the choices of decision makers.

Using the same task as in our first study, we sought to demonstrate that trials with a low probability would cancel the framing effect, because low probabilities prevent participants from anticipating a gain. On the contrary, trials with a high probability of gain should lead to the usual framing effect because they enable the positive emotional mechanism of gain anticipation. In line with Cheung and Mikels (2011), we expected that such a positive emotion would be linked to the choice of the risky option in the loss frame. More specifically, we expected that the evaluation of the participants’ emotion using a Likert-type scale would show a specific link between positive emotion and the choice of the risky option in trials with a high probability of gain, but not with a low probability of gain.

### Method

#### Participants

Forty-eight undergraduate students (24 women, mean age = 20.82; *SD* = 2.74) from the University of Paris VIII Saint-Denis were randomly assigned to the Emotion or to the Control group.

#### Material and Procedure

For both groups, the task used was the same as in the first study, but it included 40 trials according to a 5 (Initial amount: 10 vs. 20 vs. 30 vs. 40 vs. 50€) × 2 (Frame: Gain vs. Loss) × 4 [Probability of gain: Low probabilities (20% and 40%) vs. High probabilities (60 and 80%)] within-subjects design. Please note that, following the categorization of Miller and Fagley (1991), we grouped probabilities of 20 and 40% as low probabilities, because they both offer more chances of losing than winning, and probabilities of 60 and 80% as high probabilities, because they both offer more chances of winning than losing. Initial amounts ranging from 10 to 50€ were chosen because they are known not to affect the framing susceptibility of participants (Habib et al., 2015; Osmont et al., 2015). In the Emotion group, the task included a measure of the emotion felt for each trial. As recommended by Cheung and Mikels (2011), in order to assess immediate emotions, participants had to rate their emotional status just before making their choice. This was adapted from Nielson et al. (2008) and asked “How do you feel about this decision?”. Participants responded on a 7-point Likert-type scale ranging from -3 (very negative) to +3 (very positive). In the control condition, participants were not asked to assess their emotions.

#### Time Manipulation Check

Although our task was similar to the experimental design used by De Martino et al. (2006), a major difference was the absence of a time constraint. As commonly found in the framing literature and in order to be as naturalistic as possible, our participants did not have any response time limit, contrary to the limited response time (4 s) used in De Martino et al. (2006). Moreover, as the need to provide a rapid response is known to influence sensitivity to the framing effect (e.g., Takemura, 1992; Wegier and Spaniol, 2015), we carried out a pre-test check to investigate the specific influence of a 4-s limited response time on our framing paradigm.

In this pre-test, 40 participants (22 women, mean age = 20.40, *SD* = 2.43) were randomly assigned to one of the two conditions (Time: constrained vs. unconstrained). Participants in the unconstrained condition thus completed the same task as described above, but those in the constrained condition had to choose between the sure and risky options within 4 s.

A three-way ANOVA was conducted on the percentage of risky choices to assess the effect of Time (Constrained vs. Unconstrained) as between-subjects factor and Frame (Gain vs. Loss) and Probability of gain (High vs. Low) as within-subjects factors. This analysis revealed that neither the main effect of Time [*F*(1,38) = 0.81, *p* > 0.35], nor the Time × Frame [*F*(1,38) = 0.01, *p* > 0.90], nor the Time × Frame × Probability of gain [*F*(1,38) = 0.74, *p* > 0.35] were significant. However, as in the first study, we found a significant main effect of Frame [*F*(1,38) = 19.78, *p* < 0.001, η_p_^2^ = 0.34] and a significant Frame × Probability of gain interaction [*F*(1,38) = 16.25, *p* < 0.001, η_p_^2^ = 0.30]. Indeed, participants were affected by framing manipulation in the high probability condition (*p* < 0.001, *M*_Loss_ = 71% ± 29, *M*_Gain_ = 35% ± 32, η_p_^2^ = 0.43), but not in the low probability condition (*p* > 0.90, *M*_Loss_ = 50% ± 36, *M*_Gain_ = 47% ± 39).

Finally, the time constraint imposed by De Martino et al. (2006) does not seem to affect the framing effect.

### Results

For this second study, we will first describe results about the framing effect, then about the relation between emotions and choices.

First, in order to evaluate whether reporting emotions influenced the framing effect, a four-way ANOVA was conducted on the percentage of risky choices to assess the effects of Group (Emotion vs. Control) as between-subjects factor and Frame (Gain vs. Loss), Probability of gain (High vs. Low) and Initial amount (10 vs. 20 vs. 30 vs. 40 vs. 50€) as within-subjects factors. This Group × Frame × Probability of gain × Initial amount interaction was not significant [*F*(4,43) = 0.86; *p* > 0.45]. Neither Group × Frame [*F*(1,46) = 0.73; *p* > 0.40], nor Group × Frame × Probability of gain [*F*(1,46) = 0.03; *p* > 0.85] interactions were significant. However, there was a significant main effect of Frame [*F*(1,46) = 13.40, *p* < 0.01, η_p_^2^ = 0.23], indicating that participants made more risky choices in the loss frame (*M*_Loss_ = 56% ± 27) than in the gain frame (*M*_Gain_ = 46% ± 27). Then, as in our first study, the interaction of Frame × Probability of gain was significant [*F*(1,46) = 12.55, *p* < 0.01, η_p_^2^ = 0.21], indicating that participants demonstrated a framing effect in the high probability (*p* < 0.01, *M*_Loss_ = 62% ± 28, *M*_Gain_ = 43% ± 29, η_p_^2^ = 0.27) but not in the low probability condition (*p* > 0.95, *M*_Loss_ = 51% ± 29, *M*_Gain_ = 49% ± 28). Neither the main effect of the Initial amount [*F*(4,43) = 1.38; *p* > 0.25] nor the Initial amount × Frame interaction [*F*(4,43) = 0.37; *p* > 0.80] were significant.

Second, in order to determine the potential link between the evaluation of participants’ emotion and their choices (sure vs. risky option), a Generalized Estimating Equation (GEE; Liang and Zeger, 1986; Diggle et al., 1994) was carried out. GEE is an extension of the generalized linear model to repeated measures data and controls for within-cluster correlation in regression models with binary outcomes. As such, this analysis takes into consideration repeated measures of binary outcomes (i.e., all trials performed by each participant) and was thus particularly appropriate analysis for our data (Cheung and Mikels, 2011). Based on the Quasi-Information Criterion, which is a measure of the relative goodness-of-fit for GEE, an exchangeable working correlation structure was used (Pan, 2001; Burnham and Anderson, 2002). This correlation structure assumes non-zero homogeneous within-subject correlations across choices (Heck et al., 2012). In our binomial logit model, emotion was the predictor variable and choice the dependent variable. Treating emotion as a categorical (negative; neutral; positive) or continuous variable did not influence the pattern of findings. Thus, for purposes of clarity, analyses presented here were performed with emotion as a categorical variable. The results showed that emotion was not related to choice in the gain frame [χ^2^ (1, *N* = 480) = 0.13; *p* > 0.90]. In contrast, emotion was related to choice in the loss frame [χ^2^ (1, *N* = 480) = 7.39, *p* < 0.05], in that positive emotion was related to the risky choice (β = 0.71, *p* < 0.05).

**FIGURE 3:**
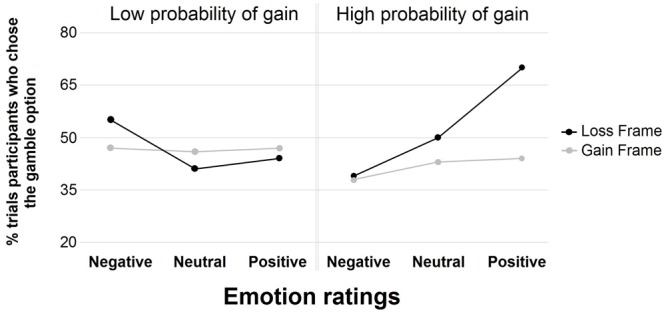
**Percentage of trial participants who chose the gamble option in the gain and loss frames with a high and low probability of gain for negative, neutral, and positive emotion ratings**.

Lastly, in order to study the modulation of the link between emotion and choice according to low and high probabilities, the same GEE as described above was carried out in the gain and loss frames but low and high probability conditions were separated (see **Figure 3**). In the high probability condition, emotion was related to the choice in the loss frame [χ^2^ (1, *N* = 240) = 10.74, *p* < 0.01], in that positive emotion was related to the risky choice (β = 2.01, *p* < 0.01), whereas emotions were not related to choice in the gain frame [χ^2^ (1, *N* = 240) = 0.68, *p* > 0.70]. In the low probability condition, emotion was not related to choice in the loss frame [χ^2^ (1, *N* = 240) = 1.07, *p* > 0.55], nor in the gain frame [χ^2^ (1, *N* = 240) = 1.33, *p* > 0.50].

## General Discussion

The objective of this article was to define precisely the role of the probability of gain in the framing effect and to understand the origin of this influence. Broadly in line with the Venture Theory, our first study showed that trials with a high but not with a low probability of gain led to the framing effect. This result was true regardless of whether participants could compare trials against each other, i.e., in the within-subjects design, or not, i.e., in the between-subjects design. Our study is the first to support the Venture Theory in a task similar to that of De Martino et al. (2006). However, interestingly, the most common framing task, the Asian-disease problem, led to a strong framing effect, even with low probabilities of gain (Tversky and Kahneman, 1981). This discrepancy between our task and the Asian-disease problem could stem from the formulation of the risky option, which was affected by framing manipulation in the Asian-disease problem but not in our task. In practical terms, in the gain frame of the Asian-disease problem, the risky option presents the probability that “all people will be saved” and the probability that “no one will be saved.” In the loss frame, the risky option presents the probability that “all people will die” or that “no one will die.” In contrast, the risky option of our task presented the probability of “keeping” or “losing” the entire initial amount for both the gain and loss frames. According to the Fuzzy-Trace Theory (FTT; Reyna and Brainerd, 1991, 1995) and supported by the experimental findings of Kühberger and Tanner (2010), the formulation of the risky option is an important condition for the emergence of the framing effect. The FTT is a cognitive process model arguing that decision-making, and more generally reasoning, is mainly driven by intuitive information processing. Based on the Asian-disease problem, FTT posits that in the gain frame, the representation of the sure option is to “save some people” while the representation of the risky option is to “possibly save none.” Thus, people would be risk-averse in the gain frame because they would find that saving some people for sure is more attractive than the possibility of saving none. In contrast, FTT posits that in the loss frame, the sure option is represented as “some people will die” and the risky option as “possibly no one will die.” Thus, people would be risk-seeking in the loss frame because they would find that some people dying for sure is less attractive than the probability that none will die. As in our task the risky option was not affected by framing manipulation, this “some-none” contrast was prevented. It is therefore likely that this methodological difference is responsible for the differences observed between our results and those of the Asian-disease problem. This hypothesis also explains the intriguing results reported by Miller and Fagley (1991), which showed that low probabilities led to the framing effect while high probabilities canceled it. As a matter of fact, these authors did not report the type of task they used and we could hypothesize that their surprising results arise from the use of an atypical formulation of the risky option.

In our second study, results replicated the influence of the probability of gain on the framing effect found in the first study. Furthermore, in line with Cheung and Mikels (2011), we confirmed that positive emotion was related to the choice of the risky option in the loss frame but not in the gain frame. However, interestingly, such a relationship between positive emotion and risky choices was only observed for the high probability condition of the loss frame. According to the model of Loewenstein and Lerner (2003), please note that our design focused on the emotions that participants actually felt just before their decisions, i.e., on immediate emotions. Nevertheless, it would be interesting in future research to separate the roles of each of the emotional influences described by Loewenstein and Lerner (2003) in the framing effect.

Moreover, our results showed that emotion was not related to choice in the gain frame, which could be due to higher emotional sensitivity toward losses than toward gains (Tversky and Kahneman, 1991). Since losses arouse stronger emotions than gains, participants probably did not discriminate their emotions for gains as clearly as they did for losses. One possible way to improve our understanding of the emotions underpinning choices in the gain frame would be to undertake a study that separates presentation of the sure and risky options and that combines a subjective and a psychophysiological measure of emotions (Yechiam et al., 2014).

Our study should also provide further insight into the discrepancy between studies focusing on the impact of emotion induction on the framing effect. Considering only financial studies investigating the influence of positive mood induction on the framing effect, four found contrasting results (Druckman and McDermott, 2008; Liu et al., 2010; Cassotti et al., 2012; Stanton et al., 2014). First, it was shown that when participants are placed in a positive emotional context, they increase their risk propensity in the gain frame, thus canceling their framing susceptibility (Druckman and McDermott, 2008). In contrast, although Cassotti et al. (2012) also found that a positive emotional context canceled the framing effect, they showed that this suppression results from a decrease in risk-taking in the loss frame. Besides, two studies reported that the induction of a positive emotional context maintains susceptibility to the framing effect but increases risk propensity in both frames. Discrepancy probably arises from the use of different probabilities. In fact, while Cassotti et al. (2012) used low, medium and high probabilities, Stanton et al. (2014) used low and high probabilities, Druckman and McDermott (2008) used only a medium probability and Liu et al. (2010) did not report which probabilities they used. Therefore, our results suggest that further investigations on emotion induction should separate high and low probability conditions, each involving different emotional mechanisms, to improve our understanding of the specific influence of positive emotion on the framing effect.

Furthermore, our results provide experimental support for decision-making models that integrate the relationship between positive emotion and risk-seeking. For example, Romer and Hennessy (2007) developed a model predicting that positive emotion is related to risk-seeking in adolescence. In fact, although adolescent risk-seeking is commonly explained by a decrease in inhibitory control (e.g., Tarter et al., 2003; Whelan et al., 2012), it has been widely demonstrated that emotions play a critical role in adolescents’ specific risk-seeking behavior (e.g., Casey et al., 2008). It is noteworthy that although this model was designed for adolescents, it was applied to adult risky decision-making. In their model, Romer and Hennessy (2007) argued that positive emotion, associated with risky behavior, would increase the likelihood of engaging in that risky behavior. Overall, our findings in the loss frame support this model. However, our results in the gain frame and in the loss frame with a low probability of gain enable us to specify this prediction. First, our results in the gain frame, with both high and low probabilities of gain, suggested that when a sure alternative to the risk seemed valuable, positive emotion was not linked to risky behaviors. Second, trials in the loss frame with a low probability of gain revealed that when the risk appeared too high, positive emotion was no longer associated with the risky choice. It would thus be interesting to investigate the emotions that underpin the framing effect in a developmental study with children, adolescents, and adults. We could thereby find out whether a specific relationship between emotion and risk-seeking, and thus the framing effect, appears during adolescence. Exploring the emotions underpinning the framing effect could improve our understanding of adolescent risk-seeking behaviors and thus provide further insight into different ways of improving specific interventions, which may help avoid the dramatic consequences of risk-seeking in adolescence (Reyna and Farley, 2006).

In the future, studying emotions that underpin the framing effect could contribute to a better understanding of everyday decisions. Although our study was confined to non-naturalistic financial decisions, the framing effect has been shown to occur in many everyday life situations. For example, the framing effect can moderate the decision to purchase green electricity (Nilsson et al., 2014), the judgment of liability when reading a newspaper story (Lotto et al., 2014), or even a crucial medical choice for one’s child (Haward et al., 2008). A better understanding of the role of emotions in the framing effect could foster a more effective formulation of public health messages, for example.

## Conclusion

Our studies have confirmed that the framing effect, one of the most robust decisional biases, can completely disappear with the simple use of a low probability of gain. It would appear that the positive emotional mechanism of gain anticipation is responsible for the variation in the framing effect according to the probability of gain. In trials with a low probability of gain, participants were no longer affected by framing manipulation because the risky option did not enable them to anticipate a gain. Finally, whether to improve the formulation of public health messages or prevent adolescent risky behaviors, it is important to pursue investigations on the emotions that underpin the framing effect.

## Ethics Statement

The study was supported by Le conseil d’evaluation ethique pour les recherches en sante (CERES). Prior to the investigation, every participants gave their written and informed consent. No vulnerable populations were involved.

## Author Contributions

The authors (CG and SM) made equal and substantial contributions to the conception, design, acquisition, analysis, and interpretation of the data.

## Conflict of Interest Statement

The authors declare that the research was conducted in the absence of any commercial or financial relationships that could be construed as a potential conflict of interest.

## Footnotes

1 A low probability of 30% was chosen for two reasons. First, it replicates the probability used in the initial paradigm of the Asian-disease problem (Tversky and Kahneman, 1981), which is one of the most common tasks used in framing research. Second, in recent years, the framing task of De Martino et al. (2006) has been increasingly used and contains 20 and 40% as low probabilities of gain. Therefore, 30% is also in the range of probabilities used by De Martino et al. (2006). Complementarily, 70% was selected as the high probability of gain.
