# Supplementary figures and images for: High But Not Low Probability of Gain Elicits a Positive Feeling Leading to the Framing Effect
Source: Front Psychol. 2017 Feb 9;8:81. doi: 10.3389/fpsyg.2017.00081 (PMC5298953; doi:10.3389/fpsyg.2017.00081)

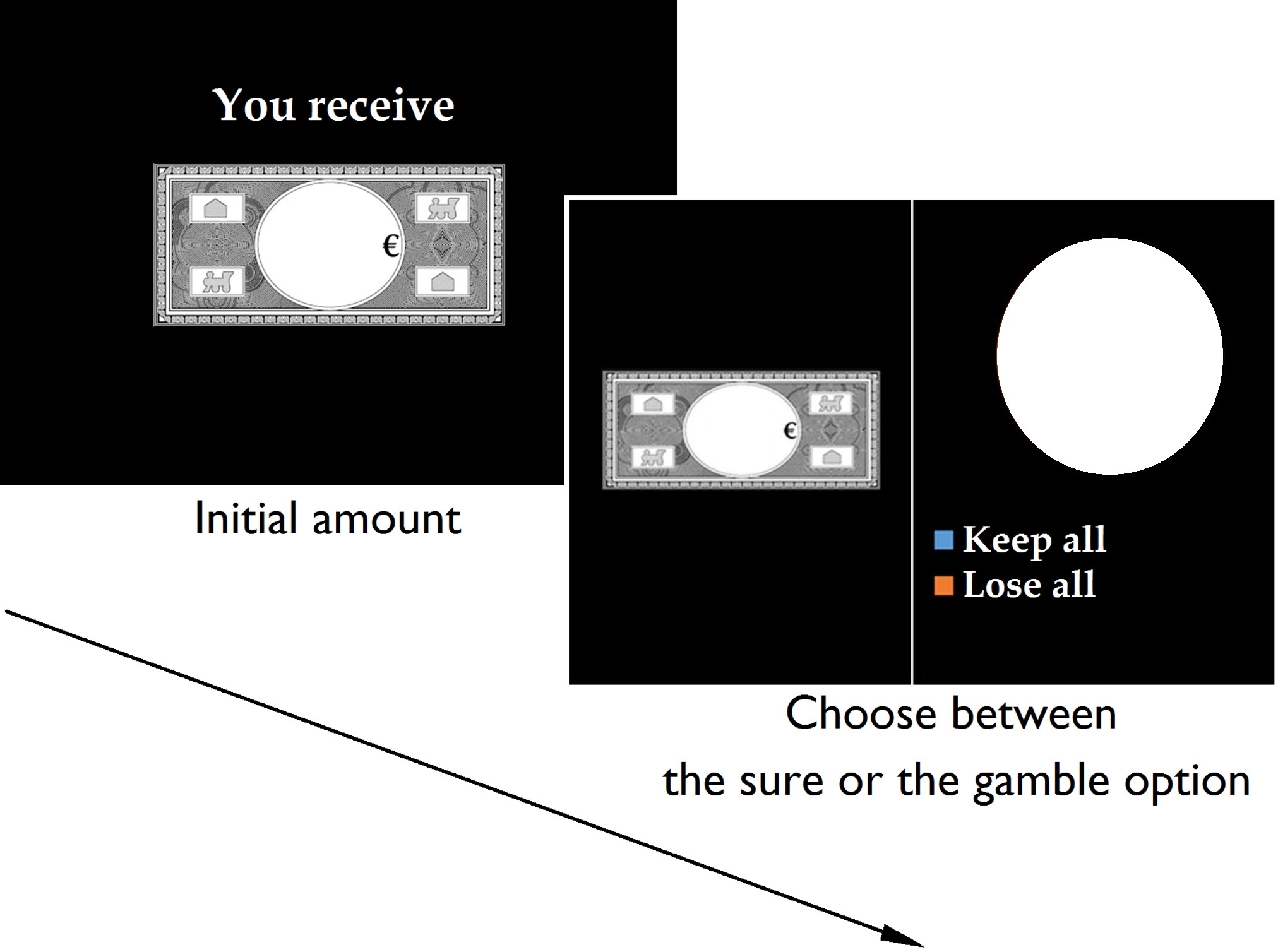

Supplement: Supplementary file 1 [file Image_1.jpg]
